# Multiple Processes Regulate Long-Term Population Dynamics of Sea Urchins on Mediterranean Rocky Reefs

**DOI:** 10.1371/journal.pone.0036901

**Published:** 2012-05-11

**Authors:** Bernat Hereu, Cristina Linares, Enric Sala, Joaquim Garrabou, Antoni Garcia-Rubies, David Diaz, Mikel Zabala

**Affiliations:** 1 Departament d’Ecologia, Universitat de Barcelona, Barcelona, Spain; 2 National Geographic Society, Washington, D.C., United States of America; 3 Centre d’Estudis Avançats de Blanes, Consejo Superior de Investigacions Cientificas, Blanes, Spain; 4 Institut de Ciències del Mar, Consejo Superior de Investigacions Cientificas, Barcelona, Spain; 5 Centre Oceanogràfic de Balears, Instituto Español de Oceanografía, Palma de Mallorca, Spain; National Institute of Water & Atmospheric Research, New Zealand

## Abstract

We annually monitored the abundance and size structure of herbivorous sea urchin populations (*Paracentrotus lividus* and *Arbacia lixula*) inside and outside a marine reserve in the Northwestern Mediterranean on two distinct habitats (boulders and vertical walls) over a period of 20 years, with the aim of analyzing changes at different temporal scales in relation to biotic and abiotic drivers. *P. lividus* exhibited significant variability in density over time on boulder bottoms but not on vertical walls, and temporal trends were not significantly different between the protection levels. Differences in densities were caused primarily by variance in recruitment, which was less pronounced inside the MPA and was correlated with adult density, indicating density-dependent recruitment under high predation pressure, as well as some positive feedback mechanisms that may facilitate higher urchin abundances despite higher predator abundance. Populations within the reserve were less variable in abundance and did not exhibit the hyper-abundances observed outside the reserve, suggesting that predation effects maybe more subtle than simply lowering the numbers of urchins in reserves. *A. lixula* densities were an order of magnitude lower than *P. lividus* densities and varied within sites and over time on boulder bottoms but did not differ between protection levels. In December 2008, an exceptionally violent storm reduced sea urchin densities drastically (by 50% to 80%) on boulder substrates, resulting in the lowest values observed over the entire study period, which remained at that level for at least two years (up to the present). Our results also showed great variability in the biological and physical processes acting at different temporal scales. This study highlights the need for appropriate temporal scales for studies to fully understand ecosystem functioning, the concepts of which are fundamental to successful conservation and management.

## Introduction

Sea urchin abundance can determine the composition, structure and persistence of benthic communities in temperate seas, which can be dominated either by large macroalgae or overgrazed communities [1]–[4]. Understanding the processes that regulate sea urchin populations is therefore crucial for revealing the mechanisms responsible for maintaining community structure or causing regime shifts. Top-down control by predatory marine vertebrates and invertebrates has been proposed as the major factor controlling algal communities in some temperate locales [5]–[10], although the evidence suggests only weak top-down control in others [4], [5], [11]–[16].

Physical factors, such as upwelling, water temperature [17], sedimentation [14], [18], [19], wave action [13], [14], [19]–[21], floods [22]–[24] and harvesting [25], [26], can also determine sea urchin abundance. In addition, low-frequency disturbances, such as mass mortality events caused by disease outbreaks, can reduce sea urchin populations for decades after the disturbance [20], [22], [27]–[32]. Other anthropogenic stressors can have interactive effects on temperate reefs, such as the harmful algae blooms that are becoming increasingly important drivers of variation in urchin populations [33]–[35].

All of the above processes may act simultaneously and on different time scales, ranging from years to decades. Thus, a better understanding of the processes and factors controlling sea urchin populations on the appropriate spatial and temporal scales is a key requirement for the effective management and conservation of subtidal temperate marine ecosystems. Since most studies encompass relatively limited spatial and temporal scales [6], [13], [14], the dynamics of sea urchin populations at large spatial scales and especially over long temporal scales are still poorly understood.

In Mediterranean nearshore rocky reefs, *Paracentrotus lividus* (Lamarck) and *Arbacia lixula* (L.) are the most common sea urchins [36]. By grazing, these species can modify the structure and dynamics of benthic communities by eliminating the canopy of perennial erect algae, inducing the formation of communities dominated by fast-growing, opportunistic species and, at high densities, inducing the formation of coralline barrens [5], [37]–[41]. Although many studies have focused on the processes that determine the population dynamics of these species, most have described the cascading effects caused by overfishing ([5], [13], [42] and references therein). Until now, a lack of long-term studies has prevented the study of low-frequency disturbances or the role of climate change in sea urchin dynamics.

In this study, we monitored sea urchin populations over 18 years to analyze their dynamics at different spatial and temporal scales. We also tested the effects of predation by comparing populations inside and outside of a Marine Protected Area. Finally, we evaluated the effects of an exceptionally strong storm that occurred along the Catalan coast in the winter of 2008.

## Materials and Methods

### Study Sites and Sampling Method

**Figure 1:**
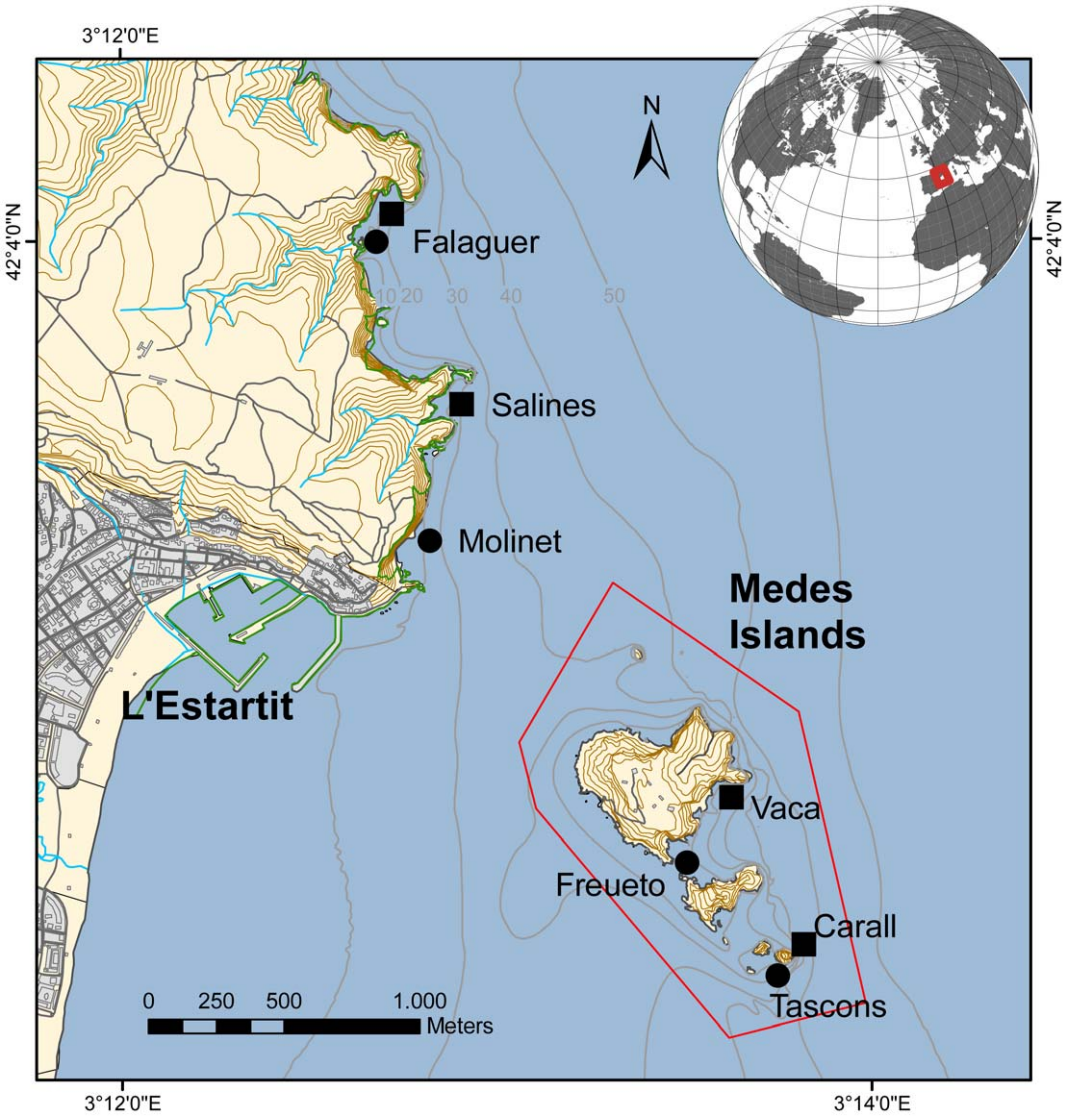
Study site. Medes Islands Marine Reserve. Locations of study sites inside (Tascons, Freueto, Vaca and Carall) and outside (Falaguer, Molinet, and Punta Salines) the reserve. The red line represents the limits of the Marine Reserve, where all fishing is prohibited.

The Medes Islands Marine Reserve (hereafter MIMR), where fishing has been prohibited since 1983 [43], is located one kilometer offshore, opposite the town of L’Estartit (NE Spain, NW Mediterranean Sea). This reserve occupies a total area of 93.2 ha and includes a group of small islands (total surface area <20 ha) (Figure 1). For years, fish populations within the MIMR have been higher in abundance and more diverse compared with nearby coastal waters outside of the reserve [43]–[50]. Sea urchin predator densities vary within the Reserve. Garcia-Rubies and Zabala (1990) [43] reported higher predator abundances on exposed shallow habitats, and Sala and Ballesteros (1997) [51] reported different abundances and habitat preferences for *Diplodus sargus* and *Doplodus vulgaris*, where *D. sargus* preferred surge and shallow zones with boulder bottoms whereas *D. vulgaris* exploited deeper waters.

While high densities of sea urchin predator species (*D. sargus* and *D. vulgaris*) were reported inside the MIMR between 1990 (9.5 to 52.16 Ind/100 m^2^; 3500 g/100 m^2^) and 2004 (3400 g/100 m^2^), lower densities of *Diplodus* species were reported in 1990 (4.8 to 13.6 Ind/100 m^2^; 800 g/100 m^2^) and in 2004 (680 g/100 m^2^) outside the MPA in the same studies [43], [52]. In addition, predation rate was demonstrated to be higher inside the reserve through several tethering studies in the area [47], [49], [53].

Within this reserve, sea urchin populations were monitored annually for 18 years from its creation in 1991 to 2010, with a gap between 2006 and 2007 because of logistical constraints. To assess the effect of fishing pressure on fish predators on sea urchin populations, two sites inside the MIMR and two nearby sites on the non-protected coast were selected [54]. Furthermore, to assess the role of topography in determining the structure of sea urchin populations through the accessibility of refuges, two different types of substrate were selected: large limestone boulders (Tascons and Freuetó, within the reserve; Falaguer and Molinet, outside the reserve), and vertical walls without apparent spatial refuges (Carall and Vaca, within the reserve; Punta Salines and Falaguer, outside the reserve) (Fig. 1). The boulder habitats were colonized by a rich algal assemblage dominated by erect algae, articulated calcareous algae and small filamentous algae [55], [39], [56] (Appendix S1). The vertical walls supported the same algal assemblages as well as numerous suspension feeders, mainly small hydrozoans [39], [55] (Appendix S1).

The abundance and population structure of *P. lividus* were studied by SCUBA diving along three transects (50 m×1 m each) at a 6 m depth at each study site for each type of substrate. Transects were divided into five 10 m^2^ subtransects, and within each transect, *P. lividus* >1 cm in diameter were counted and their diameters (test without spines) were measured with a caliper. For analysis, the diameters were grouped into size classes with intervals of 1 cm and individuals were grouped into subtransects of 10 m^2^. In 1995, *A. lixula* was added to the census; this species was monitored from 1995 to 2010 at the same sites and using the same methodology as was applied to *P. lividus*. *A. lixula* is common in this area though it is less abundant than *P. lividus*, unlike in other southern areas of the NW Mediterranean.

Sampling was performed each year in the late summer to avoid possible effects of seasonality in our data. This period was selected to facilitate sampling because at this point in the year, the erect seasonal algae have disappeared and *P. lividus* shows recruitment pulses [5], [57], [58], allowing the detection of 1-year-old individuals within the study transects.

In December 2008, a severe easterly storm occurred off the Catalan coast (NW Mediterranean) with winds surpassing 85 km/hour and waves over 7 m in significant height and up to 14.4 m in maximum height. No other comparably violent storm events had been recorded in the previous 50 years. This storm had profound impacts on benthic communities at depths of up to 20 m. On boulder bottoms, large stone blocks (>3 m in diameter) were found displaced or turned upside down at depths up to 10 m causing a substantial loss of benthic cover from abrasion and erosion. Not only were algal communities denuded, but encrusting organisms, such as the date mussel *Lithofaga lithofaga*, were also affected [59]. MIMR and the nearby coast were affected by this storm, so the effects of this low-frequency event on sea urchin populations were also evaluated. After the storm, sea urchin recruits (<1 cm) were counted because we suspected that post-settlement mortality at the early stages could be important in determining the recovery of adult populations.

The level of sea urchin harvesting in this region is low, and thus, we hypothesize that the amount of harvesting did not change during the study period and that the differences over time between areas may be caused by differences in other variables such as predatory fish abundance.

Part of this work (from 1991 to 2008) was included in the Medes Islands Marine Reserve monitoring program; thus, all necessary permits for the described field studies were obtained from the authority responsible for this Marine Reserve and the nearby non-protected coast (Departament de Medi Natural, Generalitat de Catalunya). Field studies did not involve endangered or protected species, and no animal or plant was damaged.

### Data Analysis

#### Adult populations

The data were analyzed beginning in 1995, when the experimental design (sites and replicates) was standardized. To test for the effects of time, protection and habitat and the interactions between these factors on the density and mean size of *P. lividus* and *A. lixula* at each site, we performed a multiple factor ANOVA with all data obtained from 1995 to 2010 (with a gap from 2006–2008) for the 2 sites inside and outside the reserve and for each type of substrate. In this analysis, the site (random factor) was nested within the level of protection (fixed factor; protected or unprotected) and within the type of habitat (fixed factor; boulder substrates and vertical walls) to account for differences between areas and types of habitat. We included Time as a factor with 13 years, considering the measures independent over time due to the large reef area sampled each year (150****m^2^) which would diminish the potential non-independence of samples through time. Moreover, this analysis design is analogous to a split-plot design which can be used as an alternative to repeated measures [60].

The existence of a negative correlation between *P. lividus* and *A. lixula* densities, which would indicate competition between the species, was also tested using a single correlation between MIMR data for each year and site from 1995–2005.

#### Recruitment

To compare the density of sea urchin recruits (<1 cm) among the sites, types of substrate and protection levels after the storm occurred in 2008, we utilized the same ANOVA design for the data obtained in 2009 and 2010.

To test the ratio of the variances in inter-annual variability of sea urchin densities between the levels of protection on the two habitats (boulder bottoms and vertical walls), we used Fisher’s F-test.

To determine whether density-dependent juvenile survival could be operating in natural populations, we applied least-squares regression to our survey data to test for a positive association between the abundance of juveniles ≤20 mm in diameter (the average size of 1-year-old urchins; 61) in each transect and the abundance of adults ≥20 mm. Adult density was regressed against recruit density from the following year. Juvenile and adult abundances were log-transformed to improve the distribution of residuals [62].

In all data sets, the homogeneity of the variance was tested before analysis (Cochran’s test). Whenever necessary, the data were transformed with the function log (x+1). When data were not homogeneous after transformation, we reduced the level of significance to p<0.001. When statistical testing showed significant differences for the interaction, further analyses of the main effects were performed using the Tukey HSD multiple comparison test.

## Results

### *Paracentrotus Lividus*

**Figure 2:**
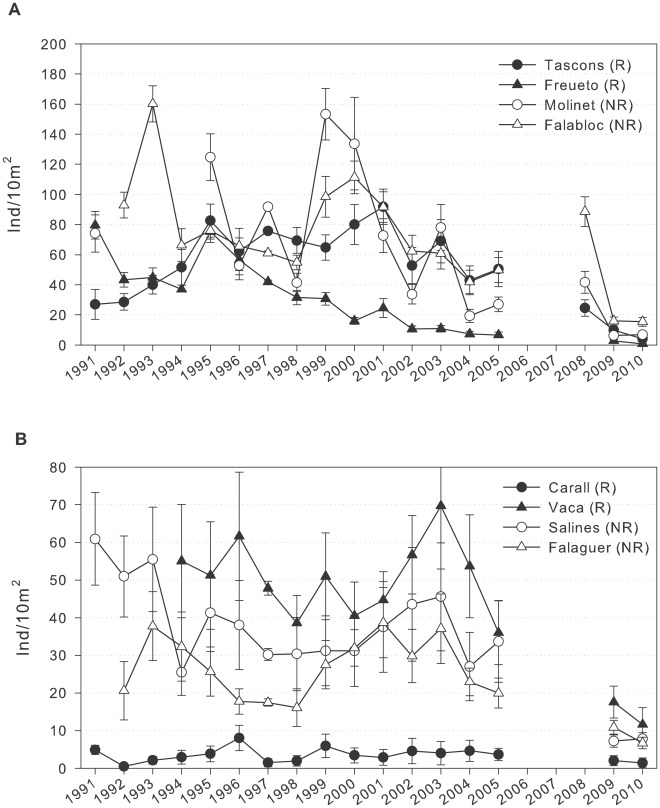
*Paracentrotus lividus* density over time. Number of *Paracentrotus lividus* (>1 cm diameter) per 10 m^2^ (mean ± SE) over time at each site in MIMR on a) boulder substrates and (b) vertical walls. Solid symbols represent sites within the reserve (R); open symbols represent sites in the nearby unprotected area (NR). Note the different scales on both types of habitat.

*P. lividus* was the most abundant sea urchin species in the MIMR with highly variable densities, especially on boulder bottoms**.** *P. lividus* densities showed a significant interaction between the sites and years, indicating significant differences in density among years at each site. There were significant differences between the substrate types over time, where urchin densities on vertical walls were lower and less variable than on boulder bottoms (Table 1). However, mean density did not differ between the protected and the unprotected area, nor was there an interaction between habitat type and protection (Table 1, Figure 2).

**Table 1:** Results of the nested ANOVA comparing the densities of *Paracentrotus lividus* between protection levels (reserve vs. non reserve), habitats (boulders vs. vertical walls) and sites (nested within Protection and Habitat) over time (1995 to 2005 and 2009–2010).

	df	MS	F	p
Protection	1	71690	1.01	0.370
Habitat	1	292748	4.15	0.111
Site (Protection*Habitat)	4	70521	21.29	**0.000**
Year	12	29807	8.99	**0.000**
Protection * Habitat	1	46874	0.66	0.460
Protection * Year	12	6085	1.83	0.068
Habitat * Year	12	15270	4.61	**0.000**
Site (Protection*Habitat) * Year	48	3313	2.66	**0.000**
Protection * Habitat * Year	12	4370	1.31	0.239
Error	1450	1245		

The only difference between urchin populations inside and outside the reserve was the variability of sea urchin densities over time, where Fisher’s F-test showed higher variability in *P. lividus* density in boulder substrates outside the reserve compared to both the boulder and vertical wall habitats inside the reserve (Table 2).

**Table 2:** Results of the F test comparing the variability in *Paracentrotus lividus* density between protected and unprotected areas and between boulder substrates and vertical walls.

	F	significance
Non Protected Boulders/Non Protected vertical	12.72	p<0.01
Non Protected Boulders/Protected Boulders	5.73	p<0.01
Non Protected Boulders/Protected Vertical	6.058	p<0.01
Non Protected Vertical/Protected Boulders	2.21	n.s.
Non Protected Vertical/Protected Vertical	2.10	n.s.
Protected Boulders/Protected Vertical	1.05	n.s.

*P. lividus* densities on boulder bottoms dropped dramatically in 2009 after an exceptionally severe storm in the winter of 2008. Densities on boulder bottoms dropped 82%, 84% and 59% in Falaguer, Molinet and Tascons, respectively, reaching the lowest values observed in the study period up to that point (Table 3, Figure 2a). In Freuetó, the density in 2009 dropped 56% from its 2005 level although there were no significant differences in the statistical analysis. The density declines were smaller on vertical walls, although there was a statistically significant drop of 78% at Salines (Table 3, Figure 2a,b). The size-frequency distribution shows that the larger size classes were the most affected by the storm (Appendix S2, S3).

**Table 3:** Results of the one-way ANOVA for each study site comparing the data on *Paracentrotus lividus* density for the years before and after the storm (in parentheses), and the percent change (%).

	df Effect	MS Effect	df Error	MS Error	F	p	%
Tascons (2008–2009)	1	1598.70	28	255.34	6.26	**0.018**	−59.6
Freueto (2005–2009)	1	108.30	28	29.75	3.64	0.066	−56.9
Molinet (2008–2009)	1	19253.33	28	2171.98	8.86	**0.005**	−84.7
Falabloc (2008–2009)	1	37730.94	23	439.73	85.80	**0.000**	−81.9
							
Carall (2005–2009)	1	19.20	28	20.70	0.92	0.343	−44.4
Vaca (2005–2009)	1	1498.13	28	748.92	2.00	0.168	−51.3
Salines (2005–2009)	1	3967.50	28	925.52	4.28	**0.047**	−78.6
Falaguer (2005–2009)	1	563.33	28	191.78	2.93	0.097	−45.4

Recruitment after the storm was very high in the Molinet population, in which a recruitment pulse occurred in 2010, with new individuals comprising 81% of the population (Appendix S4). The analysis of *P. lividus* recruits <1 cm abundance in 2009 and 2010 after the storm at different sites only showed a significant interaction between year and site (F_4, 224_ = 1.17, p<0.001; Appendix S4), indicating a highly variable recruitment among sites and years, with no differences between the protected and unprotected areas. Molinet was the location where recruitment was highest, with a peak in 2010, whereas we did not observe any recruitment at Freuetó (Appendix S4).

**Figure 3:**
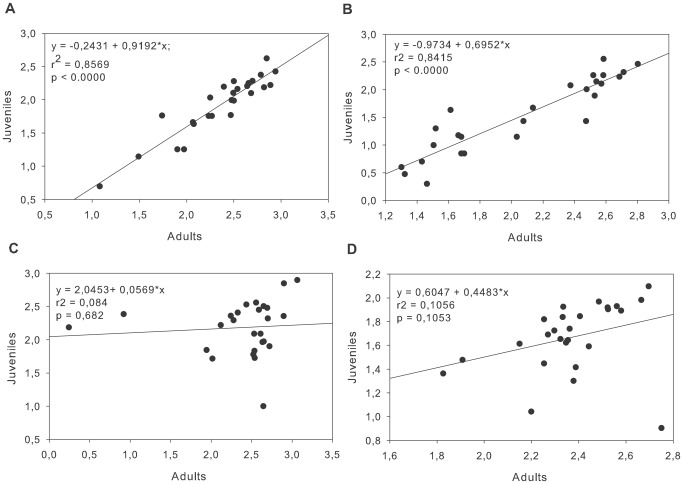
Relationship between recruits and adults. Linear relationships between recruits (diameter <2 cm) and adult *Paracentrotus lividus* in the Medes Islands Marine Reserve on a) boulder substrates and b) vertical walls and in the Montgrí coast on c) boulder substrates and d) vertical walls over the study period for log-transformed data. Each point represents the adult density at a site and the recruit abundance for the following year.

The numbers of adults (diameter >2 cm) and recruits (i.e., the number of juveniles <2 cm in the following year) in the MIMR were positively correlated on both boulder bottoms and vertical walls, with adult abundance explaining 85% and 84% of the variation in juvenile abundance, respectively (r^2^ = 0.85, df = 24 p<0.001 and r^2^ = 0.84, df = 24 p<0.001, respectively). In contrast, in the unprotected area, no relationship was found between adult abundance and juvenile recruitment (r^2^ = 0.104, df = 24 p = 0.108 and r^2^ = 0.105, df = 24 p = 0.105, respectively) (Figure 3).

On boulder bottoms, the *P. lividus* frequency-size distribution showed high variability, alternating between bimodal and unimodal distributions with conspicuous recruitment peaks (Appendix S2, S3). On vertical walls, *P. lividus* frequency-size distributions were more stable but also showed some high-recruitment episodes (Appendix S2, S3). Comparison of the mean sizes of *P. lividus* showed an interaction between time and sites, and also with substrate type, where mean size was lower on vertical walls. We also found significant interaction between protection and year (Table 4) caused by the pulses of recruitment at Molinet from 1997–2000 which reduced the mean sizes outside the reserve (Appendix S2, S4).

**Table 4:** Results of the nested ANOVA comparing the mean size of *Paracentrotus lividus* between protection levels (reserve vs. non-reserve), habitat (boulders vs. vertical walls) and sites (nested within protection and habitat), over several years (1995 to 2005 and 2009–2010).

	df	MS	F	p
Protection	1	143.7	0.2096	0.670
Habitat	1	894.2	1.30	0.316
Site (Protection*Habitat)	4	1044.6	45.08	**0.000**
Year	12	123.2	6.45	**0.000**
Protection * Habitat	1	639.8	0.93	0.388
Protection * Year	12	39.0	2.04	**0.037**
Habitat * Year	12	72.5	3.79	**0.000**
Site (Protection*Habitat) * Year	48	32.0	12.11	**0.000**
Protection * Habitat * Year	12	15.2	0.79	0.651
Error	44162	2.6		

### *Arbacia Lixula*

**Figure 4:**
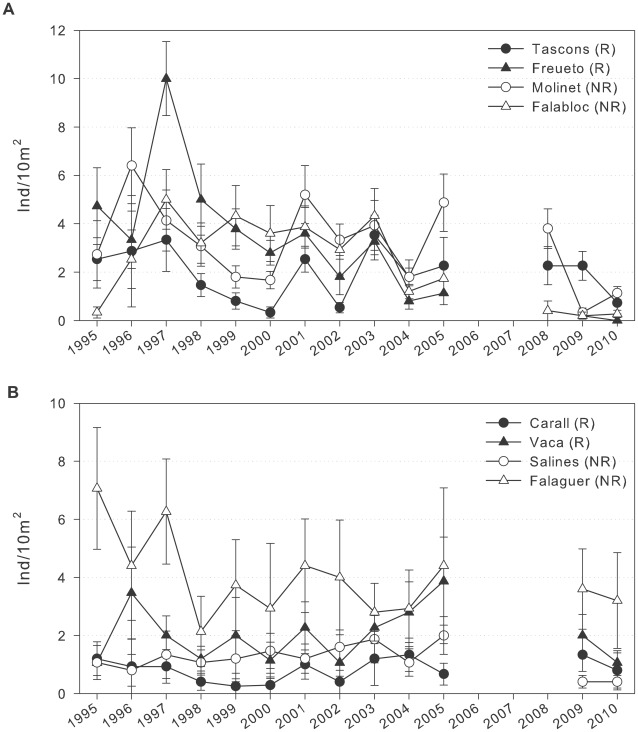
*Arbacia lixula* density over time. The number of *Arbacia lixula* (>1 cm diameter) per 10 m^2^ (mean ± SE) over time at each site in the MIMR on a) boulder substrates and (b) vertical walls. Solid symbols represent sites within the reserve (R); open symbols represent sites in the nearby unprotected area (NR). Note the different scales of both types of habitat.

Densities of *Arbacia lixula* in the MIMR were an order of magnitude lower than densities of *Paracentrotus lividus* (Figure 4) and were differently distributed, occupying more shaded and vertical habitats.

*A. lixula* densities showed a high variability over years within each site, as shown by the interaction in the analysis. The analysis also showed an interaction between substrate type and time, but no effect of the level of protection (Table 5).

**Table 5:** Results of the nested ANOVA performed on log-transformed data comparing the densities of *Arbacia lixula* between protection levels (reserve vs. non-reserve), habitats (boulders vs. vertical walls) and sites (nested within protection and habitat) over several years (1995 to 2005 and 2009–2010).

	df	MS	F	p
Protection	1	0.226	0.87	0.363
Habitat	1	0.519	2.01	0.175
Site (Protection*Habitat)	7	0.982	5.70	**0.000**
Year	12	0.745	4.03	**0.000**
Protection * Habitat	1	0.049	0.19	0.668
Protection * Year	12	0.200	1.07	0.394
Habitat * Year	12	0.552	2.97	**0.002**
Site (Protection*Habitat) * Year	57	0.203	2.14	**0.000**
Protection * Habitat *Year	12	0.294	1.58	0.118
Error	1435	0.094		

*A. lixula* densities also dropped dramatically in 2009 after the exceptionally severe storm in the winter of 2008. The densities on boulder bottoms dropped 82%, 91% and 50% at Freueto, Molinet and Falaguer, respectively, although significant differences were only found at Molinet, most likely due to the low number of individuals (Table 6; Figure 4). In contrast, urchin densities at Tascons remained constant. Density declines were more variable on vertical walls, with a decrease at Salines of 80%, a significant difference, decreases of 48% and 18% at Vaca and Falaguer, respectively, and an increase at Carall that was not significant due to the low number of individuals counted (Table 6; Figure 4). Because juveniles for this species were scarce, the majority of these declines were for adult individuals, and no recruitment was observed after the storm event (Appendix S5, S6).

**Table 6:** Results of the one-way ANOVA performed on log-transformed data for each study site comparing the data on *Arbacia lixula* density for the years before and after the storm (in parentheses), and the percent reduction (%).

	df Effect	MS Effect	Df Error	MS Effect	F	p	%
Tascons (2008–2009)	1	0.008	28	0.125	0.06	0.795	−0
Freueto (2005–2009)	1	0.179	28	0.053	3.35	0.077	−82
Molinet (2008–2009)	1	1.837	28	0.069	26.29	0.000	−91
Falabloc (2008–2009)	1	0.001	23	0.031	0.062	0.804	−50
							
Carall (2005–2009)	1	0.042	28	0.040	1.03	0.317	100
Vaca (2005–2009)	1	0.072	28	0.097	0.73	0.397	−48
Salines (2005–2009)	1	0.205	28	0.041	4.91	0.034	−80
Falaguer (2005–2009)	1	0.020	28	0.157	0.13	0.717	−18

The size structure of *Arbacia lixula* populations showed a unimodal distribution, with dominance of the 4 cm size class (Appendix S5, S6). Analysis of the mean sizes of *A. lixula* revealed a significant interaction between site and year, but no difference between the substrate types or between protected and unprotected areas was apparent (Table 7). *P. lividus* and *A. lixula* densities were not significantly correlated on either boulder bottoms or slope bare rock (r^2^ = 0.006, df = 42, p = 0.87; r^2^ = 0.037, df = 42, p = 0.209, respectively).

**Table 7:** Results of the nested ANOVA comparing the mean size of *Arbacia lixula* between protection levels (reserve vs. non-reserve), habitats (boulders vs. vertical walls) and sites (nested within protection and habitat) over several years (1995 to 2005 and 2009–2010).

	df	MS	F	p
Protection	1	3.86	0.304	0.610
Habitat	1	31.94	2.541	0.185
Site (Protection*Habitat)	4	14.02	7.213	**0.000**
Year	12	2.41	1.205	0.301
Protection * Habitat	1	0.00	0.000	0.995
Protection * Year	12	1.07	0.535	0.882
Habitat * Year	12	3.81	1.902	0.052
Site (Protection*Habitat) * Year	47	2.76	4.463	**0.000**
Protection * Habitat * Year	12	2.07	1.033	0.431
Error	2458	0.62		

## Discussion

### Top-down Control Predictions: Juvenile Mortality and Spatial Scales

In contrast with the deterministic results predicted by the top-down control theory, one of the main findings of the study was the similar densities obtained at protected and unprotected sites despite the high fish densities maintained at the protected sites throughout the study period (>10 years) [43]–[53].

Although high-settlement episodes were observed both inside and outside the MPA [62], the recruitment pulses were more conspicuous outside the MPA. This result may indicate a certain level of predation control within the MPA; predators may dampen high-recruitment episodes, stabilizing and potentially controlling sea urchin populations. These results suggest that although the high biomass of MPA fishes may make urchin population oscillations less disruptive, predation cannot fully counteract the destabilizing effects of massive larval recruitment.

Size structure patterns in sea urchin populations support this hypothesis. Populations with a high proportion of recruits are characterized by either a bimodal structure (with one mode in the adult sea urchin size range and another in the juvenile size range), which is typical of sea urchin populations under a certain degree of predation pressure (e.g., [58], [64]), or a unimodal structure in which juvenile sizes dominate because of recruitment pulses.

In the protected area, there was a high correlation between adult and juvenile abundances; this correlation did not occur in the non-protected area. Because recruitment is not site-selective [46], these results suggest that juvenile survival is density-dependent and facilitated by adults when predation pressure is high. We hypothesize that this effect could be a consequence of both the transformation of the microhabitat around adult sea urchins and the protection provided by adult sea urchins. *Paracentrotus lividus* modify the algal substrate around themselves, most likely clearing the substrate of turf and sediment and also preventing the presence of micropredators, which could increase the survival of juveniles, as demonstrated for other species (e.g., [65]–[67]). Additionally, adult sea urchins can exert a level of protection over juvenile sea urchins [68]–[71]. When sea urchins are placed on small crevices or irregularities, more microrefuges are created (as small cavities between the adults and the rock) where juvenile sea urchins and other characteristic fauna such as ophiurids (*Ophitrix fragilis*, *Ophiocomina nigra*), some endolithic sponges (*Cliona viridis*) or clingfish (*Lepadogaster lepadogaster*) can take refuge (authors’ personal observation).

In contrast, when predation pressure is low, juvenile survival is independent of adult populations as recruits can survive in more open microhabitats. Pulses of recruitment, especially in the non-protected area, explain the change in population size structure from a bimodal to a unimodal structure, as described above.

Other bimodal distributions in the size structure of sea urchin populations are also attributed to size-dependent predation pressure and the effects of recruitment [58], [72]–[74]. In central California kelp forests, some *Strongylocentrotus franciscanus* populations exhibited a bimodal size distribution caused by density-dependent juvenile mortality because juveniles are protected by the spine canopy of adults. A lower predation rate for adults over a threshold value in size was also described [64], [72].

A scaling effect may explain the poor forecasting ability of trophic cascade models. The MIMR is relatively small and is separated from the non-protected study sites by only one kilometer of the nearby coast, which encompasses many square kilometers of rocky sea bottom occupied by dense sea urchin populations. Given the long planktonic life of sea urchin larvae [75] and the capacity for passive dispersion by coastal currents, the population of the MIMR is a very small part of a metapopulation that displays highly active demographic interchange. Genetic studies of this species have shown low structure in *P. lividus* populations, suggesting high gene flow between populations [76]. Thus, even if the fishes were capable of greatly depressing the reproductive subpopulation of the MPA, they cannot prevent recruitment pulses, which are heavily supplemented by external subpopulations.

Nevertheless, although there was no overall effect of the reserve on density, our results indicate that the highest densities were consistently recorded at fished sites. If predators have the greatest effect on juveniles and therefore recruitment into the adult population, over time they may limit adult density. In a recent review, Babcock et al [77] analyzed a long-term time series of ecological data at several MPAs and demonstrated that indirect effects based on trophic cascades can take more than a decade to develop. In the Leigh marine reserve, the delayed effect of predators took >15 years to control urchin densities [6]. After 17 years (1991–2008), this lagged effect has not been clearly observed inside MIMR. Nevertheless, low-frequency strong disturbances (such as the storm that occurred in December 2008) may in fact accelerate this process by reducing urchin densities to a level where predators are then able to control their densities. Given the density-dependent survival of recruits observed in fished sites and the high juvenile predation rate inside the reserve [47], the abundance of urchins in the reserve following the storm may not recover to the original densities, and recovery is likely to be slower than at fished sites.

The other evidence of predation control was the cryptic behavior of sea urchins. In a parallel study, Sala [78] demonstrated that sea urchins were more cryptic inside the marine reserve. Other studies of sea urchin behavior performed in this area have demonstrated that movement and home range are lower inside the marine reserve due to the presence of fish predators, thus reducing the grazing effect on algal communities [79], [80]. Similar situations exist in other reserves, where densities may be similar inside and outside of reserves but there are behavioral differences [33].

Beyond the effects of predation, a significant factor in determining sea urchin population structure and density was the topography. Differences between populations on boulder bottoms (with higher densities and an abundance of small individuals) and vertical walls (with lower densities and dominance by adult individuals) were maintained throughout the study period. The absence of refuges on vertical walls causes higher predation of juveniles, resulting in dominance by adults; the size structure is thus bimodal and less affected by recruitment pulses. In contrast, on boulder bottoms with a high availability of refuges, juvenile mortality is density-independent, with a high proportion of juveniles and frequent changes in size structure from bimodal to unimodal. On boulder bottoms outside the MIMR, changes in size structure are more frequent than inside the MIMR because of high refuge availability and a lower predation rate (see comments above).

At a small spatial scale, differences in densities among sites within areas of both habitats suggest that differences in microhabitat features, settlement rate, or fish predator rate might exist at a scale of hundreds of meters. Hereu et al. [63] reported significant differences in the recruitment rates at scales of tens of meters. Likewise, fish densities are different among sites within the reserve [45]–[53], which can result in different predation rates. The topography could also be an important factor in explaining differences between the sites. The sites were selected for similar substrates, sizes of boulders, orientation, and water motion, but disregarded differences in microhabitat (such as microshelters) might result in differences in the survival rate of recruits.

The exceptionally severe storm that occurred in December 2008 caused heterogeneous changes in benthic communities within the study area, which depended on the orientation and substrate type. Because the waves moved northwest, the Montgrí coast was the most affected site. Based on parallel studies [59], we estimated 76% and 38% losses of algal cover on the Montgrí coast and Medes Islands, respectively. These losses were proportional to the loss of sea urchin density and biomass. This was the most important storm-related mass mortality episode in the sea urchin populations. Other acute low-frequency perturbations, such as mass mortality caused by disease, have been described for other temperate and tropical sea urchin species with long-term consequences for the whole ecological community (e.g., [14], [28], [81], [82]). In the Caribbean, more than 93% of the black sea urchin *Diadema antillarum* populations were lost in 1983, causing regime shifts from corals to macroalgae ([37] and references therein), and the population remained at less than 10% of its original density after more than 20 years. In our study, profound changes in *P. lividus* population structure were recorded two years after the event. Despite the recruitment pulse observed in 2010 at some sites, we believe that the affected populations will not recover for many years because of their relatively low growth rate [61] and the limited migration capacity of this species [79]. Because the predation pressure on juveniles is higher inside the marine reserve and the recruitment pulses there are more attenuated compared to the non-protected area, we predict that the recovery of sea urchin populations inside the reserve will be slower than outside the reserve.

Other environmental factors can interact with predators and modify the effects on sea urchin populations. For example, in New Zealand a large bloom of the toxic algae *Ostreopsis siamensis* enhanced predation rates on sea urchins due to sublethal effects, thus leading to greater divergence in sea urchins densities between fished sites and unfished sites where predators were more abundant [33]. All of this evidence, together with the present study, suggests that acute low-frequency perturbations, such as diseases or storms, can effectively control sea urchin populations. These disturbances not only decimate sea urchin populations but may change their dynamics and the intensity of the processes that regulate them, such as recruitment and predation.

### The Role of *Arbacia Lixula*

Although *A. lixula* and *P. lividus* co-occur on hard substrata in shallow subtidal habitats and their competitive relationship has been discussed (e.g., [83]–[85]); in our long-term study, we found no clear relationship between *A. lixula* and *P. lividus* abundance. These results agree with Pais et al. (2007) [85], who found only moderate competition for habitat and resources between these two echinoids after analyzing the impact of heavily harvesting *P. lividus* populations from sea urchins communities on shallow rocky reefs. We conclude that, in the studied areas, *A. lixula* dynamics are not determined by the abundance of *P. lividus* populations and are most likely more strongly influenced by factors other than competition. While *P. lividus* is more common on horizontal and photophilic habitats (feeding mainly on fleshy algae and suspended organic particles), *A. lixula* is more abundant on shaded vertical substrata and overhangs, preferring encrusting corallines [37], [83]–[87].

*A. lixula* populations remained very low throughout the monitored years, with densities approximately 1/10 those observed for *P. lividus*. In contrast with *P. lividus*, *A. lixula* showed no conspicuous oscillations in density, and size structure remained constant, with a high frequency of large size classes and very low recruitment, which is rarely observed along the northwest Mediterranean coast (this study, [57], [63], [86], [88]). This species is considered thermophilic [86], and its abundance can vary by orders of magnitude depending on the region, suggesting a biogeographical pattern (e.g., [40], [86], [89]).

Because of the thermophilicity of the species, it has been suggested that *A. lixula* abundance is affected by the increase in temperature caused by climate change [89]–[93]. Our long-term data do not support this hypothesis. Despite the warming of coastal waters by nearly 1°C over the past 3 decades in the northwest Mediterranean sea [93], [94], the *A. lixula* populations here have not undergone conspicuous change after 15 years, with densities lower than those reported in Scandola by Francour et al. [90].

### The Lack of Appropriate Study Scales: Implications for Conservation and Management

The results of this study highlight that not only predator effects but also processes acting at different temporal and spatial scales (from local and annual to regional and low-frequency) can modify the generally linear processes that regulate sea urchin populations. Transitions between alternate states (e.g., macroalgal beds and barrens) could be driven by critical thresholds, not only in the abundance of predatory fish [7] but also in sea urchin densities, which, in turn, are regulated by factors other than predation [3], [5], [6], [14], [17]–[35].

Some studies have demonstrated that top-down control by predators is context-dependent and will vary depending on local physical conditions and on the characteristics of species that are locally dominant [13], [14]. Indirect effects (i.e., trophic cascade effects) on benthic communities are also mediated by many processes that can delay their appearance, such as the delays in direct effects, or the characteristics of the indirect responses themselves [77]. In the Medes Islands, direct effects on fish populations were described after less than 5 years of protection [43]. Although fish predator densities were maintained above 15 ind/100 m^2^ (the threshold predicted to be needed to control sea urchin densities; [7]), differences on sea urchin populations were not highly conspicuous after more than 15 years of protection. A similar time lag has been observed in several temperate and tropical reserves, where sea urchin predators increased rapidly but the effects of predators on herbivore and algal community abundances took more than a decade to develop [81]. This lag was explained by the sheltering behavior of sea urchins that reduces the effects of predation. In the Medes Islands, sheltering behavior [53], together with trait-mediated reductions in sea urchin grazing [80], could also explain the moderate indirect effects of fishing on sea urchin populations and their effects on algal communities.

In a recent study, Sala et al. [48] studied several MPAs and non-protected areas in the Mediterranean and did not find a clear effect of protection on benthic algal communities. Most of the largest recorded biomasses of *Cystoseira* canopies, which are considered an indicator of “healthy” rocky reefs [95]–[97], were found at unprotected sites, indicating that factors other than fishing are largely responsible for the structure of Mediterranean benthic communities. Medes Islands, one of the oldest reserves in the Mediterranean, is the only location in which a recovery of *Cystoseira* spp. canopy was observed after protection [39], [48], [55], suggesting that the recovery of formerly abundant *Cystoseira* canopies in the NW Mediterranean [96] takes longer than the recovery of fish assemblages. In general, it has been shown that indirect effects take considerably longer than direct effects [77].

Our results show high levels of variability in the biological and physical processes controlling sea urchin populations. We find that not only physical factors but also low-frequency extreme events are important. Only long-term monitoring programs with regular periodicity can integrate the effects of regulating factors that act at different temporal scales. Long temporal scales are needed to avoid misinterpreting processes or confounding factors. In contrast, short-term studies may attribute population trends to inappropriate causes, such as fish predation or climate change. Long-term studies, well-designed and regularly performed, are fundamental to understanding the functioning of natural ecosystems, as such studies provide evidence that cannot be detected by short-term experimental or space-for-time substitution studies [77]. The coupling of experimental and long-term descriptive approaches is desirable for understanding ecosystem functioning; experimental studies should be used for testing and investigating processes, but long-term series are needed to observe the ways in which these processes and factors interact in nature.

## Supporting Information

Appendix S1 **Major benthic algal species in the study assemblage. Species are pooled into two groups for data analysis: ^a^ seasonal, ^b^ perennial.**
(DOC)

Appendix S2 ***Paracentrotus lividus*** **(>1 cm) frequency of each size class from 1991 to 2005 on large boulders within (Tascons and Freuetó) and outside (Molinet and Falaguer) the Medes Islands Marine Reserve.** Size classes: 1 = 1−1.9 cm, 2 = 2−2.9 cm, 3 = 3−3.9 cm, 4 = 4−4.9 cm, 5 = 5−5.9 cm, 6 = 6−6.9 cm, 7 = 7−7.9 cm.
(TIF)

Appendix S3 ***Paracentrotus lividus*** **(>1 cm) frequency of each size class from 1991 to 2005 on slope bare rocks within (Carall and Vaca) and outside (Salines and Falaguer) the Medes Islands Marine Reserve.** Size classes: 1 = 1−1.9 cm, 2 = 2−2.9 cm, 3 = 3−3.9 cm, 4 = 4−4.9 cm, 5 = 5−5.9 cm, 6 = 6−6.9 cm, 7 = 7−7.9 cm.
(TIF)

Appendix S4 **Number of** ***Paracentrotus lividus*** **(<1 cm diameter) per 10 m^2^ (mean ± SE) on 2008, 2009 and 2010 at each site in Medes Islands Marine Reserve and nearby non-protected Montgrí coast a) in boulder bottoms and (b) slope bare rocks.**
(TIF)

Appendix S5 ***Arbacia lixula*** **(>1 cm) frequency of each size class from 1991 to 2002 at 6 m depth on large boulders within (Tascons and Freuetó) and outside (Molinet and Falaguer) the Medes Islands Marine Reserve.** Size classes: 1 = 1−1.9 cm, 2 = 2−2.9 cm, 3 = 3−3.9 cm, 4 = 4−4.9 cm, 5 = 5−5.9 cm, 6 = 6−6.9 cm, 7 = 7−7.9 cm.
(TIF)

Appendix S6 ***Arbacia lixula*** **(>1 cm) frequency of each size class from 1991 to 2002 at 6 m depth on slope bare rocks within (Carall, Vaca) and outside (Salines, Falaguer) the Medes Islands Marine Reserve.** Size classes: 1 = 1−1.9 cm, 2 = 2−2.9 cm, 3 = 3−3.9 cm, 4 = 4−4.9 cm, 5 = 5−5.9 cm, 6 = 6−6.9 cm, 7 = 7−7.9 cm.
(TIF)
