# Supplementary material for: Multiple Processes Regulate Long-Term Population Dynamics of Sea Urchins on Mediterranean Rocky Reefs
Source: PLoS One. 2012 May 11;7(5):e36901. doi: 10.1371/journal.pone.0036901 (PMC3350477; doi:10.1371/journal.pone.0036901)
Supplement: Appendix S1 — Major benthic algal species in the study assemblage. Species are pooled into two groups for data analysis: a seasonal, b perennial. (DOC) [file pone.0036901.s001.doc]

| FAMILY | SPECIES |
| --- | --- |
| Cladostephaceae | *Cladostephus hirsutus* (Linnaeus) Prudhonne van Reine ^b^ |
| Cutleriaceae | *Cutleria adspersa* (Mertens ex Roth) De Notaris ^a^ |
|  | *Zanardinia prototypus* (Nardo) Nardo ^b^ |
| Cystoseiraceae | *Cystoseira compressa* (Esper) Gerloff et Nizamuddin ^b^ |
|  | *Cystoseira* sp. ^b^ |
| Dictyotaceae | *Dictyopteris membranacea* (Stackhouse) Batters ^a^ |
|  | *Dictyota dichotoma* (Hudson) Lamoroux ^a^ |
|  | *Dictyota fasciola* (Roth) ^a^ |
|  | *Padina pavonica* (Linnaeus) Thivy ^a^ |
| Phyllariaceae | *Taonia atomaria* (Woodward) J.Agardh ^a^ |
| Punctariaceae | *Asperococcus* *turneri* (Smith) Hooker ^a^ |
| Scytosiphonaceae | *Colpomenia sinuosa* (Mertens ex Roth) Derbis et Solier in Castagne ^a^ |
| Sphacelariaceae | *Sphacelaria cirrosa* (Roth) C. Agardh ^b^ |
| Stypocaulaceae | *Halopteris filicina* (Grateloup) Kützing ^b^ |
|  | *Halopteris scoparia* (Linnaeus) Sauvageau ^b^ |
| Bonnemaisoniaceae | *Asparagopsis armata* (Harvey) Schmitz ^a^ |
|  | *Bonnemaisonia asparagoides* (Woodward) C.Agardh ^a^ |
|  | *Falkenbergia rufolanosa* (Harvey) F.Schmitz - stadium ^a^ |
| Ceramiaceae | Ceramiaceae ^a^ |
| Corallinaceae | *Amphiroa rigida* Lamouroux ^b^ |
|  | *Corallina elongata* Ellis et Solander ^b^ |
|  | *Jania* spp. ^b^ |
|  | *Lithophyllum incrustans* Philippi ^b^ |
|  | *Mesophyllum alternans* ^b^ |
| Gelidiaceae | *Gelidium latifolium* (Greville) Bornet et Thuret ^a^ |
| Helminthocladiaceae | *Liagora viscida* (Forskål) C.Agardh ^a^ |
| Peyssonneliaceae | *Peyssonnelia* sp.^b^ |
| Plocamiaceae | *Plocamium cartilagineum* (Linnaeus) P.Dixon ^b^ |
| Rhodomelaceae | *Wrangelia penicillata* C.Agardh ^a^ |
|  | *Laurencia obtusa* (Hudson) Lamouroux ^a^ |
|  | *Polysiphonia* spp. ^a^ |
| Sphaerococcaceae | *Sphaerococcus coronopifolius* Stackhouse ^b^ |
| Acetabulariaceae | *Acetabularia acetabulum* (Linnaeus) Silva ^a^ |
| Bryopsidaceae | *Bryopsis* sp. ^a^ |
| Codiaceae | *Codium bursa* (Linnaeus) Kützing ^b^ |
|  | *Codium effusum* (Rafinesque) Delle Chiaje ^b^ |
|  | *Codium vermilara* (Olivi) Delle Chiaje ^b^ |
| Dasycladaceae | *Dasycladus vermicularis* (Scopoli) Krasser ^a^ |
| Udoteaceae | *Flabellia petiolata* (Turra) Nizamuddin ^b^ |
|  | *Halimeda tuna* (Ellis et Solander) Lamoroux ^b^ |
| Ulvaceae | *Ulva rigida* C.Agardh ^a^ |
| Valoniaceae | *Valonia utricularis* (Roth) C.Agardh ^a^ |
