# Supplementary figures and images for: Multiple Processes Regulate Long-Term Population Dynamics of Sea Urchins on Mediterranean Rocky Reefs
Source: PLoS One. 2012 May 11;7(5):e36901. doi: 10.1371/journal.pone.0036901 (PMC3350477; doi:10.1371/journal.pone.0036901)

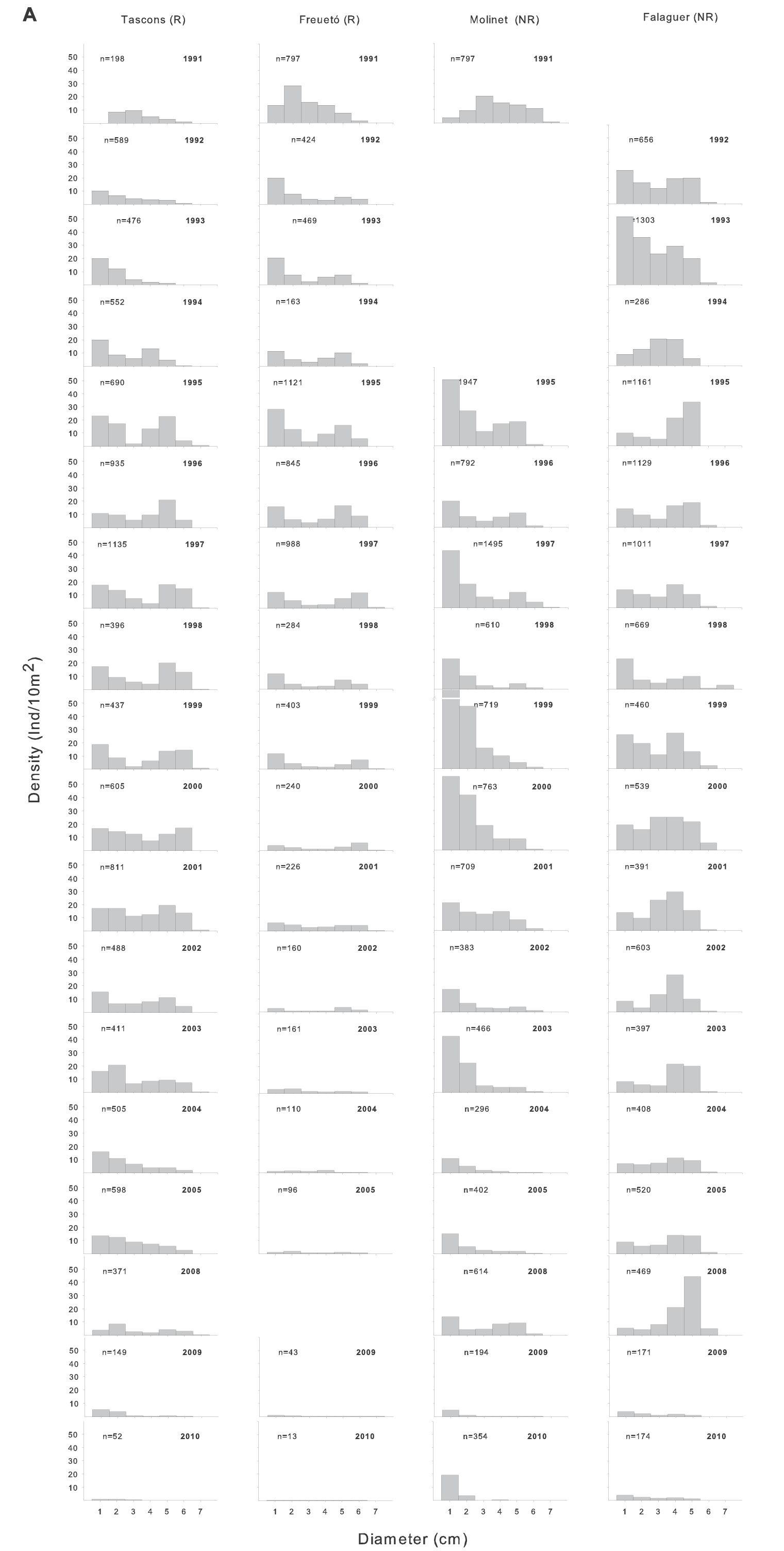

Supplement: Appendix S2 — Paracentrotus lividus (>1 cm) frequency of each size class from 1991 to 2005 on large boulders within (Tascons and Freuetó) and outside (Molinet and Falaguer) the Medes Islands Marine Reserve. Size classes: 1 = 1−1.9 cm, 2 = 2−2.9 cm, 3 = 3−3.9 cm, 4 = 4−4.9 cm, 5 = 5−5.9 cm, 6 = 6−6.9 cm, 7 = 7−7.9 cm. (TIF) [file pone.0036901.s002.tif]

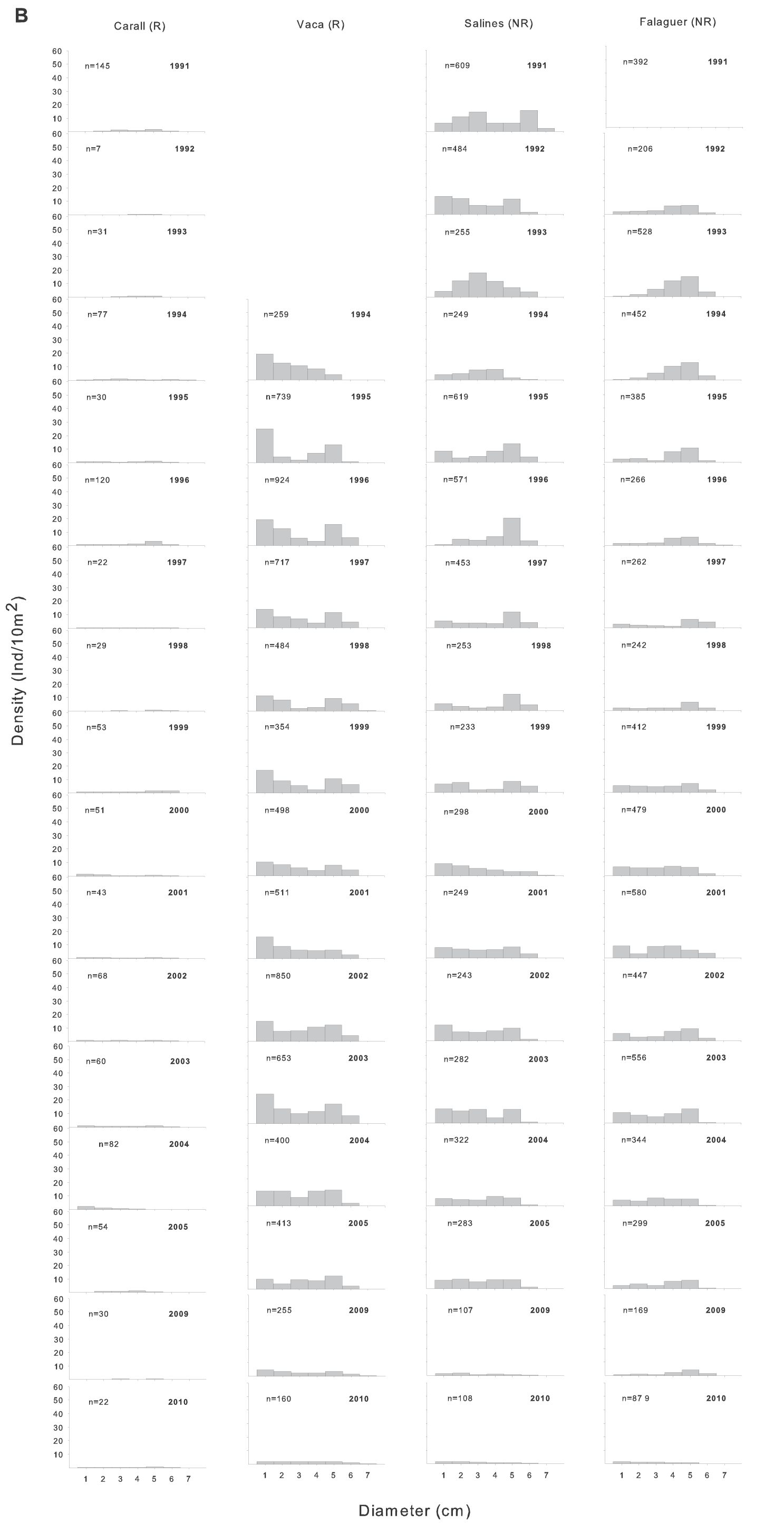

Supplement: Appendix S3 — Paracentrotus lividus (>1 cm) frequency of each size class from 1991 to 2005 on slope bare rocks within (Carall and Vaca) and outside (Salines and Falaguer) the Medes Islands Marine Reserve. Size classes: 1 = 1−1.9 cm, 2 = 2−2.9 cm, 3 = 3−3.9 cm, 4 = 4−4.9 cm, 5 = 5−5.9 cm, 6 = 6−6.9 cm, 7 = 7−7.9 cm. (TIF) [file pone.0036901.s003.tif]

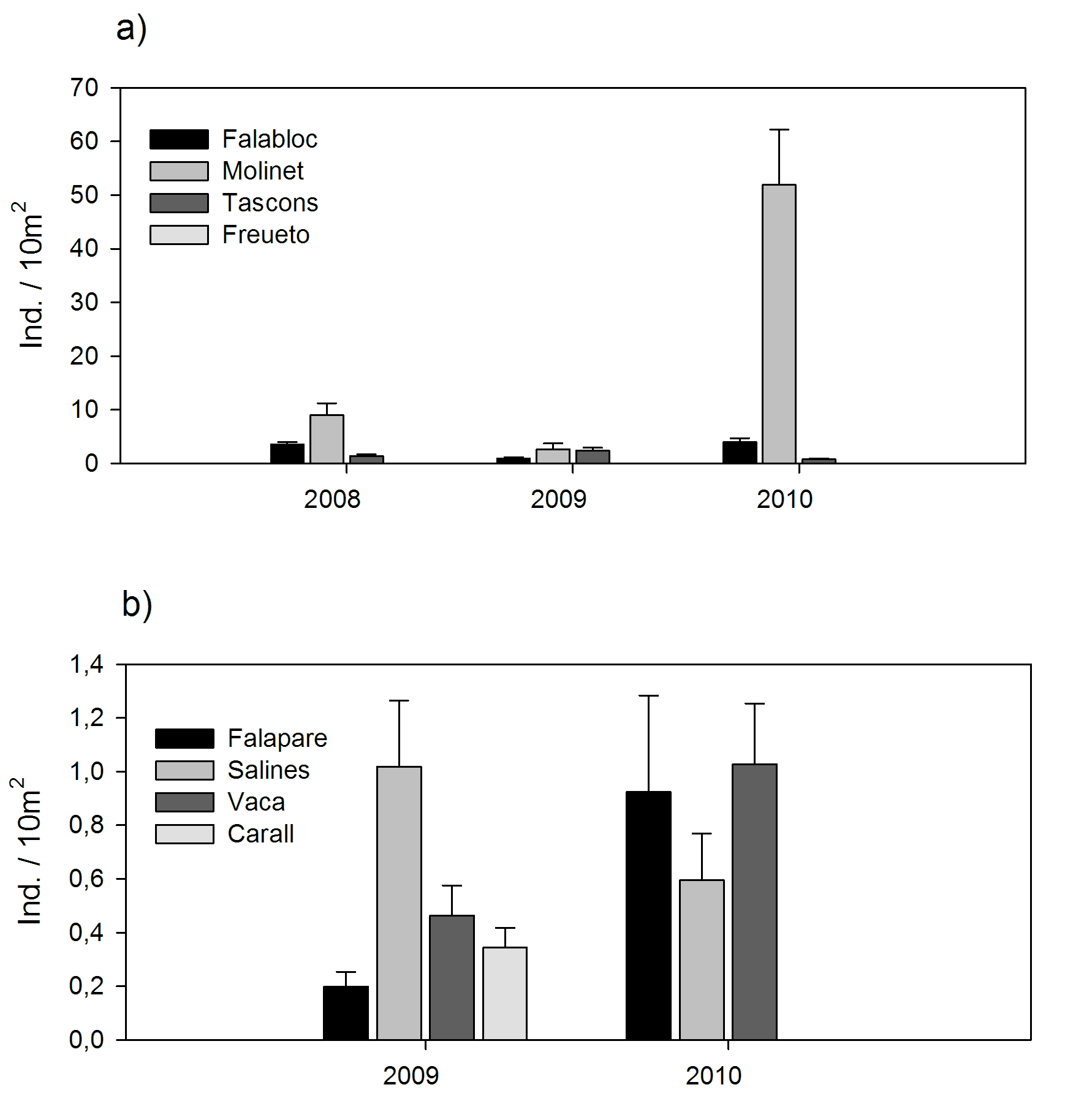

Supplement: Appendix S4 — Number of Paracentrotus lividus (<1 cm diameter) per 10 m2 (mean ± SE) on 2008, 2009 and 2010 at each site in Medes Islands Marine Reserve and nearby non-protected Montgrí coast a) in boulder bottoms and (b) slope bare rocks. (TIF) [file pone.0036901.s004.tif]

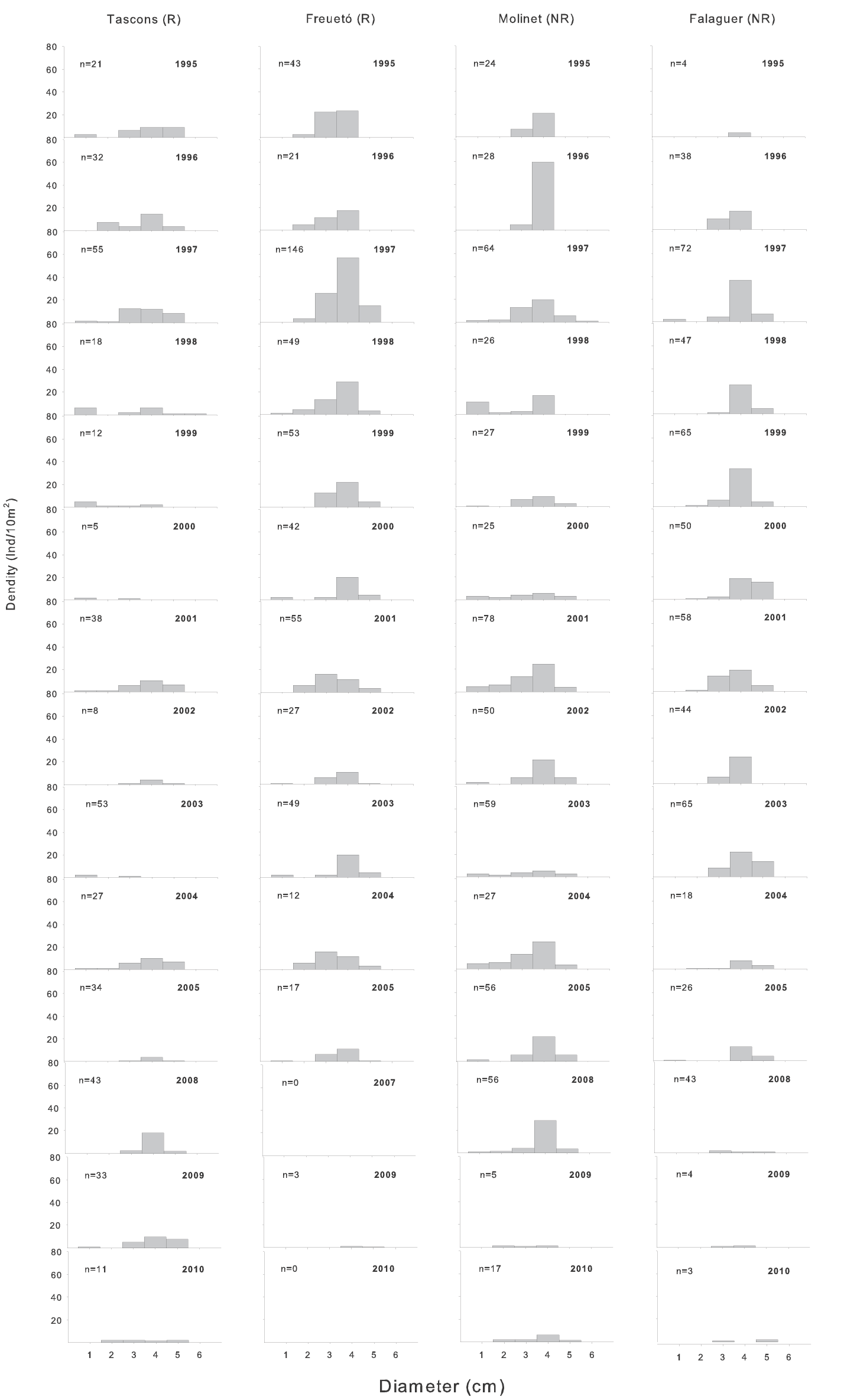

Supplement: Appendix S5 — Arbacia lixula (>1 cm) frequency of each size class from 1991 to 2002 at 6 m depth on large boulders within (Tascons and Freuetó) and outside (Molinet and Falaguer) the Medes Islands Marine Reserve. Size classes: 1 = 1−1.9 cm, 2 = 2−2.9 cm, 3 = 3−3.9 cm, 4 = 4−4.9 cm, 5 = 5−5.9 cm, 6 = 6−6.9 cm, 7 = 7−7.9 cm. (TIF) [file pone.0036901.s005.tif]

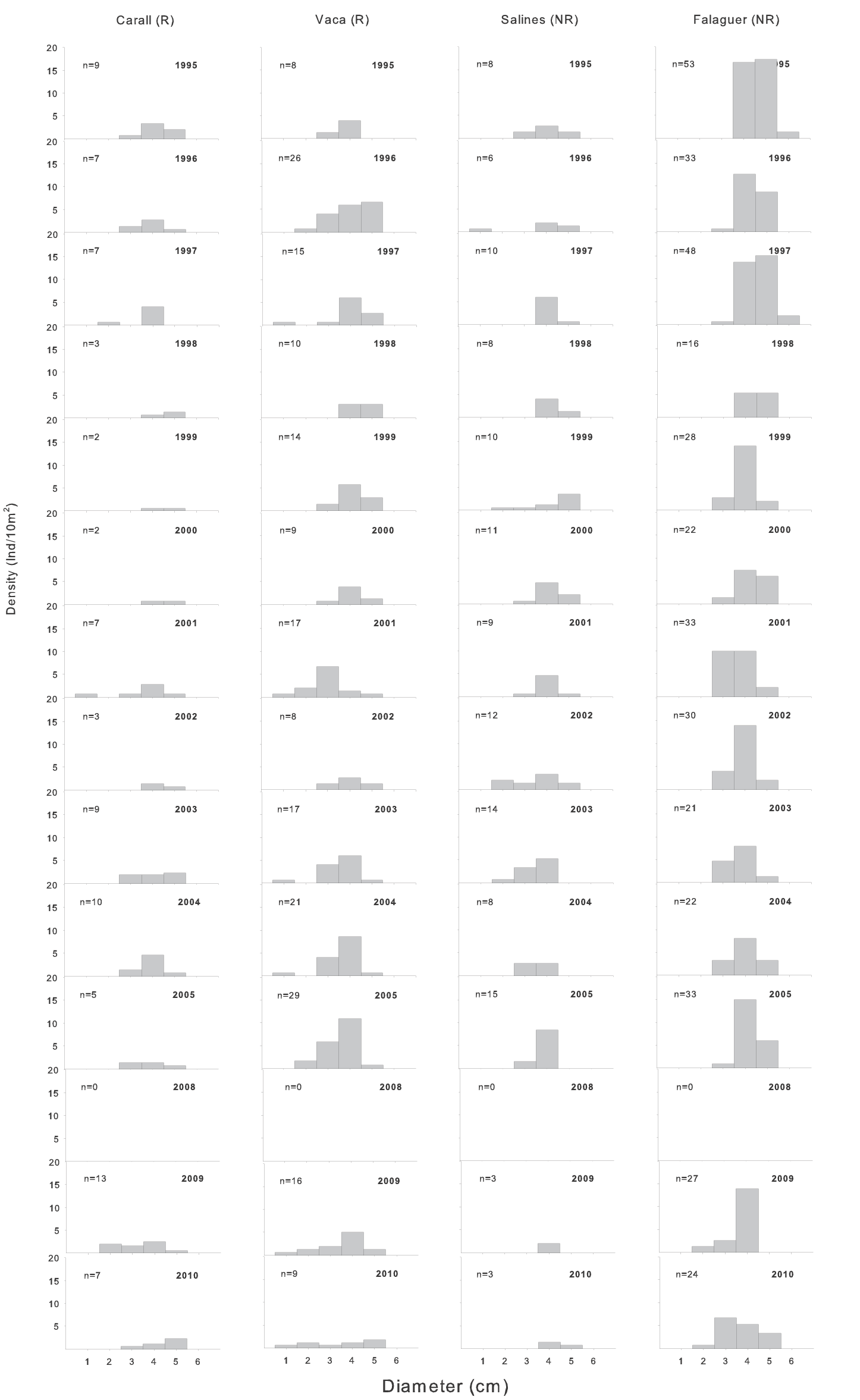

Supplement: Appendix S6 — Arbacia lixula (>1 cm) frequency of each size class from 1991 to 2002 at 6 m depth on slope bare rocks within (Carall, Vaca) and outside (Salines, Falaguer) the Medes Islands Marine Reserve. Size classes: 1 = 1−1.9 cm, 2 = 2−2.9 cm, 3 = 3−3.9 cm, 4 = 4−4.9 cm, 5 = 5−5.9 cm, 6 = 6−6.9 cm, 7 = 7−7.9 cm. (TIF) [file pone.0036901.s006.tif]
